# On the molecular basis of enduring memory in neurons, and cell fate in fibroblasts

**DOI:** 10.1093/nar/gkag337

**Published:** 2026-04-28

**Authors:** Peter R Cook

**Affiliations:** The Sir William Dunn School of Pathology, University of Oxford; Oxford, OX1 3RE, United Kingdom

## Abstract

Memories can last a lifetime, and how this is achieved remains an unanswered challenge. Most current thinking sees molecular traces of memories (engrams) as sets of synaptic proteins facilitating neuronal co-firing and co-wiring. However, most proteins turn over in months or less. Another challenge is how fibroblasts remember their cell fate for decades, and an emerging model sees functionally related genes co-firing in clusters (called transcription factories and condensates) that make RNAs specifying cell fate. As clustering is driven by entropic forces acting throughout time, the first cells may have possessed this memory system, and Nature could have exploited it to store engrams when nervous systems evolved. Then, transcription creates the naïve neuronal substrate and defines which cells are included in co-wiring and co-firing circuits, before progressive cell differentiation consolidates long-term memories. I speculate that transcription plays another central role. For every nucleotide added to a nascent RNA, transcription generates a pyrophosphate—a chelating agent that sequesters the calcium ions that can modify action-potential spike-trains. In other words, the same nano-wired DNA computer that specifies cell fate could store and manipulate our memories.

## Graphical Abstract

**Graphical Abstract:**
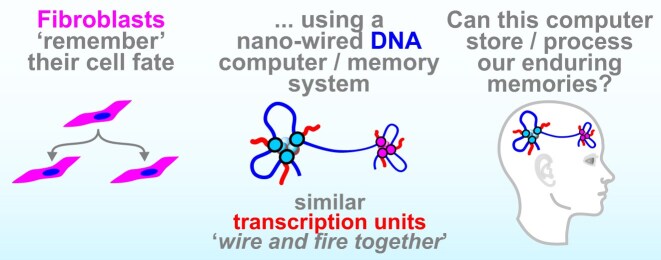

## Introduction: neural engrams

**Figure 1.**
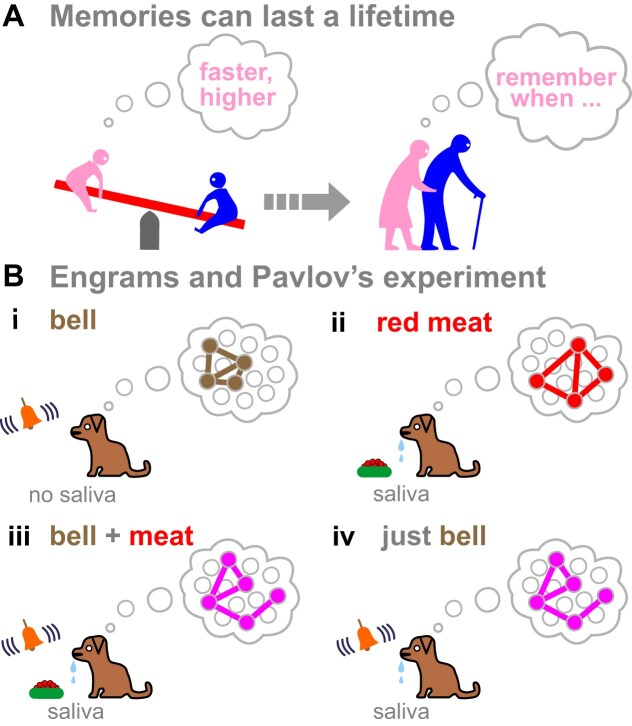
Memories and engrams. (**A**) Memories can be enduring. (**B**) Pavlov noticed a dog did not salivate on hearing a sound (a metronome or buzzer), but did salivate (through a fistula, into a tube) when presented with red meat. I follow modern practice and show a bell sounding, and free salivation. After repeatedly serving meat with an associated sound, the dog learned to salivate. Bubbles indicate hypothetical Hebbian wiring diagrams between engram cells.

Memories can last a lifetime (Fig. 1A), and providing molecular explanations for this remains a central challenge in neuroscience. More than a century ago, Semon proposed a theory of memory involving engrams [1]. Engrams have many definitions; I use the one of Josselyn and Tonegawa that sees them as enduring offline physical/chemical traces of memories (elicited by learning, and found in groups of neurons) [2]. My focus will be on traces lasting decades.

Neurons active during a memory test are often marked by phosphorylation of transcription factors (TFs) like CREB (cAMP response element-binding protein) that stimulate firing of immediate-early genes (e.g. *c-fos*); then, phosphorylated CREB or c-fos in neurons points to an engram. More persuasive evidence is obtained by disrupting function before acquisition, retrieving memories in the absence of cues by electrically stimulating engram cells, and by implanting memories without prior training to mimic related ones [2].

In what I will call the ‘standard’ model [2], a memory trace is a network of neurons with ‘strengthened’ synapses that work together, as postulated by Hebb and which is captured by ‘*cells that fire together, wire together*’ [3]. Then, proteins at synapses stabilizing connections would constitute an engram, with memories being retrieved as electrical spike trains. This is illustrated by attaching hypothetical engrams—different wiring diagrams or connectomes—to Pavlov’s dog during the famous conditioning experiment (Fig. 1B). Information travels in two general ways: through action potentials in spike trains passing down neurons (coupled to vesicular traffic across synapses), and as calcium (Ca) transients traveling within and between neurons and surrounding cells through gap junctions and nanotubes—a network generally seen as supportive of the neuronal one [4–9]. Memory processing and computation then occur at synapses in the neural net.

Long-term potentiation (LTP)—a persistent increase in synaptic strength or connectivity—underlies such wiring [10]. LTP is sub-divided depending on the molecules involved (e.g. receptor, ion channel) or time span (e.g. short-term lasting minutes to hours, early lasting hours, and late—lasting longer, and requiring protein synthesis plus transcription and proteasome-mediated degradation) [11–13]. Dendritic spines often appear over days as synaptic strength increases before shrinking, with such synaptic plasticity correlating with remembering and forgetting [14].

### Insights from hardware and software mimicking our brains

Modern computers use silicon chips with von Neumann architectures, where memory addresses are logically identical and store data or instructions. In contrast, addresses in our brains are non-equivalent, with interlinked memory and processing. Consequently, brain-like hardware [15, 16] and software [17–20] are being developed. Both have surprising properties.

Neuromorphic hardware often contains memristors (a portmanteau of ‘memory’ and ‘resistors’). Unlike a resistor, a memristor ‘recalls’ how much charge previously passed through it, even when powered off. Flows through two thermodynamically isolated memristors spontaneously evolve towards a stable (unchanging) equilibrium with maximal entropy. In contrast, thermodynamically open pairs are blessed with the possibility of generating complex emergent oscillations—but only in a precisely defined domain called ‘the edge of chaos’ [21]. In memristor networks, two measures of information flow—transfer entropy and active information storage—are maximal when the system is close to chaos [22]. This begs the question: do Hebbian networks operate at the edge of chaos?

The conceptual inventor of memristors—Chua—has provided insights into chaos theory generally (e.g. explaining the Turing instability, and resolving the Smale paradox), and how action potentials emerge [23, 24]. In the latter case, he begins with the second law of thermodynamics: axons only fire if supplied with external sources of energy. He then imagines a neuron sits at the edge of chaos (an asymptotically stable state) where it acquires the ability to amplify infinitesimally small fluctuations in energy—and so generate ‘all-or-none’ spikes. He next uses nonlinear differential equations to calculate the precise parameters defining this Goldilocks zone. His parameters reproduce those obtained experimentally, and he concludes our brains work on the edge of chaos.

Spiking neural networks (SNNs) are usually the core algorithms, where ‘spikes’ carry information and spike-timing-dependent plasticity adjusts ‘synaptic weights’. Use of non-equivalent addresses (compared to identical ones) brings many advantages [25]: neuromorphic algorithms are speedier (memory and processing are interlinked, so there is no von Neumann bottleneck), more efficient (data is processed locally, so ‘olfactory’ and ‘auditory’ ‘neurons’ can simultaneously run different algorithms), more energy saving (so ‘neurons’ do not overheat), adaptable (‘synaptic weights’ change on the fly), and more stable (decentralized networks retain function despite connection losses). Consequently, SNNs perform complex tasks like recognizing gestures and spoken commands much like humans [26]. Of course, these neuromorphic algorithms are ill-suited to the precise sequential tasks performed by our laptops, and debugging them is notoriously difficult.

### Cellular substrates of engrams

I choose granule cells as exemplars as they are the main neuronal type in the mouse cerebellum [27, 28], but the arguments apply to other cells in other layers in the net. Unsurprisingly, granule cells play central roles in brain development and function [29]. They are distinguished by having the shortest distance (∼15 µm) between nuclei and synapses of all neurons. They also express region-specific sets of messenger RNAs (mRNAs) [30]. Note that mRNAs can be speedily translocated from nuclei to synapses at ∼0.1–1 µm/min [31, 32]. Even in pyramidal cells where input and output synapses lie hundreds of microns apart, nuclei are always strategically positioned between the two [33]. Moreover, the soma of ∼5 granule cells is often covered by ‘veils’ of cytoplasm from one glial cell (a velate protoplasmic astrocyte) [4]. Why has evolution minimized distances between nuclei and functional contacts with adjacent cells?

Despite the few morphological cell types in our brains, single-cell transcriptomics uncovers a bewildering number of different types [34–37], and establishing how many there are depends on the algorithms and thresholds used [38]. I illustrate this variety using ‘fruitless’ neurons encoding reproductive behaviors of the fruit fly. Analysis of only ∼2500 cells uncovered 113 distinct groups, and detailed inspection of 46 of these showed that homeobox-containing TFs varied the most in expression, and immunoglobulin-like domains were enriched the most—with each group expressing an unique repertoire [39]. This points to TFs driving differentiation within one neuronal pathway to give many different cell types that stick together.

### Brain cells are transcriptionally hyper-active

Transcription clearly plays an important role in engram formation and function [40–42]. For example, transcriptional activity is tightly linked to depolarization and its associated Ca transients [43–45]. Moreover, adding nicotine to neuronal precursors growing *in vitro* immediately depolarizes them, increasing *c-fos* transcription tenfold in 5 min [46]. Hundreds of other genes encoding TFs are also switched on in minutes [47]. Similarly, KCl depolarization activates transcription within 2 h at > 12 000 enhancers, doubling the amount of RNA polymerase II bound to them [48]. Again remarkably, brain cells express more (different) enhancer RNAs (eRNAs) than any other tissue in the FANTOM5 mouse dataset [49], more mRNAs than fibroblasts [37], and extraordinary numbers of long non-coding, micro, and circular RNAs [50–52]. Note that nascent human eRNAs outnumber nascent mRNAs by ∼10:1 [53].

Expression patterns reminiscent of those occurring during development change during engram writing and reading. For example, neurons activated after subjecting mice to foot shock were isolated and analyzed [54]. Chromatin immediately becomes more accessible. During memory consolidation, a few genes become active as promoter:enhancer interactions reorganize. During retrieval, new contacts appear between promoters and enhancers primed during encoding, and many genes involved in local protein synthesis and synaptic morphogenesis become more active. These slow changes mirror those seen during differentiation. Another example highlights roles of particular TFs [55]. Two sets of mice were trained in a reward-associated context: one set at high repetition (so they remembered their training for weeks, detected by licking in anticipation of the reward), and the other less often (so they forgot). Transcriptomics of single cells in the thalamocortical circuit uncovered distinct groups of TFs correlating with remembering and forgetting. Targeted CRISPR-knockouts then pointed to some TFs having no effect on memory formation, as others affected stabilization (e.g. calmodulin-dependent factor CAMTA1 was required for memory maintenance for days, but TCF4 and histone methyltransferase ASH1L were needed to remember for weeks).

Note that many odd brain transcripts are derived from promoters in transposable elements that spread coincidentally with the evolution of mammalian brains [56, 57]. Additionally, the most rapidly evolving sequences in our brains are duplications of > 1 kbp that are seen as drivers of our expanded neocortex and higher neuronal connectivity; I note that 11 of the 31 candidate genes in these duplications encode surface molecules [58].

Such results beg many unanswered questions: why are neural transcriptional activities so correlated, hyper-active, and ‘noisy’ (apparently at the edge of chaos), and why can we not just scale up a fly brain to enable us to think like we do (without needing new TFs)?

### A paradox: our brains are bimodal, acting both very quickly and very slowly

Zheng and Meister [59] use broad-brush figures to highlight the stark contrast between the throughput of a human being (only ∼10 bits/s) compared to that of our sensory systems that gather data at ∼10^9^ bits/s. The smaller figure (10 bits/s) applies to all human behaviors, including perception, cognition, and motor function; it was obtained by averaging activities like speaking, reading, typing, memorizing digits, and solving Rubik’s cube. Note that messages from one neuron to the next depend on spike timing (cortical firing rates are typically a few spikes/s), and that information theory tells us there are ∼2 bits/spike. The larger throughput (10^9^ bits/s) is derived by considering cones in one eye. Each receptor converts a dynamic light input into a continuously varying membrane voltage (output ∼270 bits/s), and 6 million cones produce ∼1.6 × 10^9^ bits/s. From this, a copy-typist’s brain sifts out just the 10 bits/s needed to perform the necessary key strokes. Zheng and Meister recognize that many will be upset by their claim that our brains work so slowly (e.g. see [60]), but nevertheless insist that input and processing bitstreams are very different.

Now consider how the data capacity of our brain compares with that of its stored memories [59]. An upper bound of capacity was obtained by multiplying the number of synapses (∼10^14^) by the dynamic range of synaptic strength (∼5 bits)—which gives ≈ 50 TB. If one adds a few bits per neuron to specify different functional parameters, this hardly increases the number as there are only ∼10^11^ neurons [61]. Since this capacity of ≈50 TB is so huge, there is scope for massive redundancy and/or selection of a tiny sub-set of the data. Moreover, if one were to soak up information at 10 bits/s for 24 h a day for 100 y without sleeping, all resulting memories amount to <4 GB! As this is so small, it must mean there is massive inter-leaving of different memories (holograms provide an analogous compression of sets of spatial information) [62].

Zheng and Meister conclude our brains are bimodal: high-dimensional and peripheral inputs are dramatically filtered into sparse bitstreams at the center [59]. In other words, there are two kinds of memory: a short-term one where information is rapidly transferred between cells through synapses, and a second where data is stored and manipulated slowly. Gershman also suggests there must be two memory mechanisms using different algorithms: inference parameters are stored at synapses and updated via synaptic plasticity, while generative ones have another molecular format within individual cells that is updated biochemically [63]. In other words, the system operates different memory/processing modules (digital, analogue) at different speeds in different places.

### Some problems with synaptic memory

Despite widespread belief that synaptic connections capture long-term changes elicited by learning, many argue that direct evidence is lacking. For example, a fraction of the counter-evidence listed by Gershman is as follows [63] (see also [64]). (i) Pre- and post-synaptic neurons must fire within 40–60 ms to change synaptic strength, but animals learn associations between stimuli separated by hours. (ii) Most spines (markers of synaptic strength/connectivity) in the hippocampus are replaced every 2 weeks, but memories can last much longer. (iii) *Drosophila* larvae trained by electro-shocking to avoid an odor retain the memory through metamorphosis when relevant synapses (between olfactory-projection and mushroom-body neurons) disassemble. (iv) Hippocampal LTP is reduced in mutant mice lacking an isoform of protein kinase C, but mutants still learn to perform a hippocampus-dependent task (swimming through a Morris water maze). I add to this list: (v) Instincts and their associated analogs of engrams called ‘ingrams’ survive for generations [65]. For example, laboratory mice bred in cages scurry for cover when exposed to images of potential predators that could only have been seen generations earlier [66, 67]. (vi) Synaptic transmission is energetically expensive, but human sensory stimulation and solving cognitive problems only marginally increases energy demand. Moreover, energy costs of the brain per unit mass in primates is roughly proportional to neuronal number, not synaptic number or connectome complexity [68]. (vii) Ca transients passing through networks of nanotubes (length ∼3 μm) provide alternatives to synaptic inter-cell connectivity, but these nanotubes have half-lives of hours [8]. A feature common to much of this counter-evidence is the difficulty of imagining memory mechanisms able to last a lifetime.

## Memory molecules

**Figure 2.**
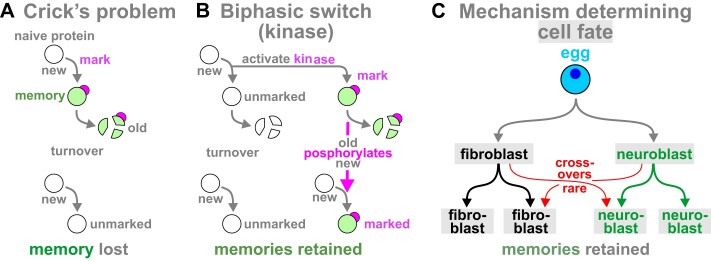
A problem faced by any memory protein, and two solutions. (**A**) Crick’s problem: if a protein is marked (purple) when it carries a memory (green), protein turnover drives memory loss. (**B**) Crick’s solution involved a biphasic switch. Here, a kinase deposits a mark (a phosphate group, purple) that enables the now-marked protein to phosphorylate newly made proteins, and this ensures the memory persists. (**C**) The system determining cell fate encodes a memory: when a fibroblast divides, progeny ‘remember’ they are fibroblasts (not neuroblasts).

DNA is arguably the best substrate for long-term information storage, and is widely used for this purpose when computing *in vitro* [69]. Sequences from sediments in North Greenland hold the current longevity record of ∼2 M years; they encode an ecosystem containing poplars, mastodons, reindeer, and geese [70]. Then, one can imagine that acquiring a memory changes a sequence much as lymphocyte DNA records a memory of an antigen; however, there is no evidence for such directed changes (but there are undirected ones) [71]. RNA is less stable, but is rarely thought to encode engrams (however, see [64]). Frances Crick pointed out that nearly all macromolecules other than DNA turn over in months or less (Fig. 2A), and suggested a simple persistence mechanism that depends on a bi-stable switch (Fig. 2B) [72]. Many such switches are known (e.g. CAMK2A and PKMζ that affect synapse stability) [12, 73]. These kinases have active (phosphorylated) and inactive states, and—when new inactive ones appear—they remain unaltered if most existing molecules are inactive. However, phosphorylation activates them so they now phosphorylate inactive ones. Consequently, new molecules replacing older ones acquire the state of older ones—and a molecular memory survives. Stability is enhanced if kinases are part of multimeric structures (CAMK2A is a dodecamer, and PKMζ is anchored to a large KIBRA complex) [74]. This mechanism survives replacement of all its parts, just as Theseus' ship persisted despite renewal of all its timbers.

Whilst proteins are popular memory molecules [2, 12, 75, 76], others continue to be suggested. These include modifications of DNA—through G4-quadraplexes [77], methylation [78, 79], or deposition of marks on associated histones [78, 80], as well as RNA—through new synthesis, m6A modification, alternative splicing, and even inter-neuron transfer of mRNAs encoding proteins like ARC, and CAMK2A [81–83]. All such mechanisms require two ‘missing links’—one transferring information from synapses to internal memory stores (nuclei in the case of histone modifications), plus a second generating responsive spike trains—all in the second it takes to recall a memory. Consequently, they have not found wide acceptance, and Crick’s biphasic switches are seen in most memory models. I now describe two very different memory mechanisms.

## Memory of cell fate and circadian rhythm

When a fibroblast divides, progeny usually ‘remember’ their cell fate for decades (Fig. 2C). This is why transcriptomics allows us to define cell types so accurately [84–87]. Such remembering is cell-autonomous, as all fibroblasts in a clone are demonstrably fibroblasts.

A memory system governing circadian rhythms is super-imposed on this cell-fate one in organisms from the simplest bilaterian to man. These rhythms involve conserved negative-feedback loops, bi-stable switches, and transcriptional activators/repressors (e.g. in man, the CLOCK–BMAL1 complex plus PER1, PER2, CRY1, CRY2 drive circadian transcription of ∼5%–20% of the genes expressed in any particular tissue) [88, 89]. While such rhythms are synchronized by the daily solar cycle (in man, through the hypothalamic suprachiasmatic nucleus), they persist in cells grown in perpetual darkness. Why are fate choices and circadian rhythms so enduring? To address this, I review how TFs regulate expression of particular gene sets specifying fate.

### An alternative view of transcription

**Figure 3.**
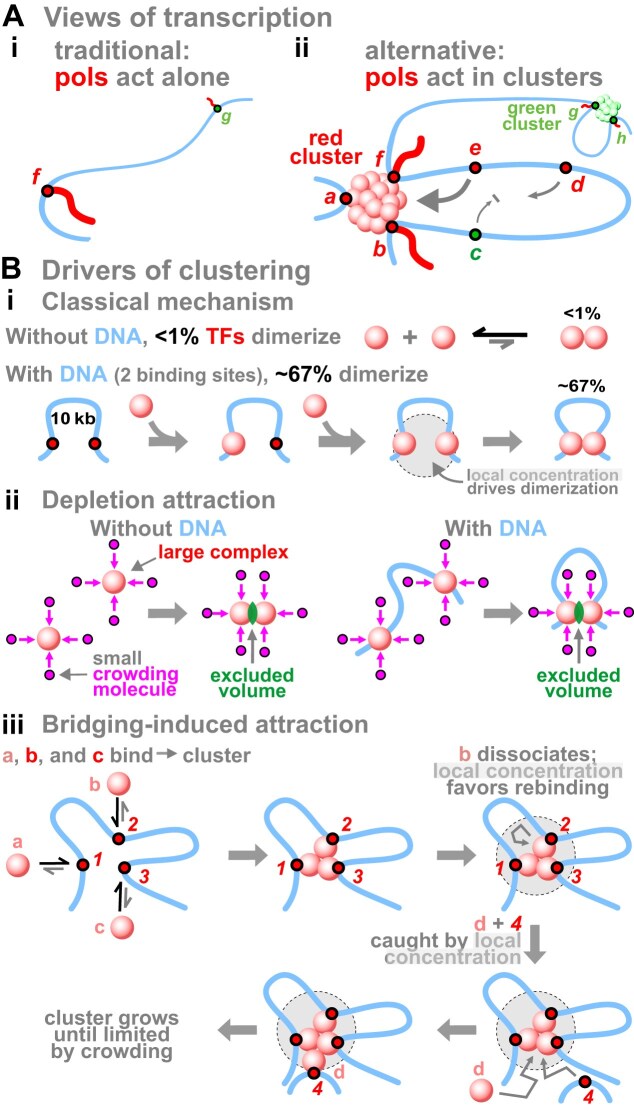
Models for transcription, and three drivers of clustering. (**A**) Models. **(i)** RNA polymerases (pols) act alone. **(ii)** We now know that clusters of polymerases make most transcripts. Here, the red cluster (rich in red TFs) transcribes red *b *+ *f*, as the green cluster (rich in green TFs) transcribes green *g *+ *h*. Unit *a* is bound to a TF in the red cluster and may soon fire. Firing frequency is largely determined by 3D distance of a promoter to a cluster rich in appropriate TFs (here, *e* diffuses to the red cluster and fires more often than *d*). *c* may visit the pink cluster as often as *e*, but does not fire there as appropriate TFs are lacking (it could fire in the distant green cluster, but cannot do so until *b* terminates). Clusters appear/disappear as TFs, polymerases, and DNA bind/dissociate. There are ∼10 active transcription units per factory in human cells [98], ∼6000 pol II clusters in mouse ES cells [171], and TNFα switches on many inflammatory-response transcription units in HUVECs with 150–250 of them initially being co-transcribed in a (small-world) group of ‘NFκB’ factories [108]. (For additional quantitative data, see Cook, PR. The pan-genomic model: 8 FAQs. https://www.petercooklab.uk/pan-genomic-model/8-faqs, accessed on 9 March 2026). (**B**) Drivers of clustering. **(i)** The classical mechanism [110]. TF concentration = 1 nM, dimerization equilibrium constant = 10^−7^ M (typical values), binding sites 10 kb apart. Without DNA, <1% TFs dimerize. With DNA, once TFs bind, close tethering creates a local concentration that ensures frequent collision and ∼67% dimerization. **(ii)** The depletion and **(iii)** bridging-induced attractions (see main text).

In the conventional model for transcription [90], genes scattered along DNA are transcribed independently of others (Fig. 3Ai). However, evidence now points to clusters of RNA polymerases making most transcripts (Fig. 3Aii) [91]. Such aggregates are called transcription factories [92, 93], clusters [94], condensates [91], drops [95], hubs [96], and pockets [97]. I will use the term ‘cluster’ for these structures, as all contain a local concentration of the machinery that works through the law of mass action to ensure efficient RNA production (e.g. the concentration of RNA polymerase II in a factory in a mammalian cell is ∼1000-fold higher than in the nucleoplasm) [92]. This view leads to a ‘pan-genomic’ model with two core concepts [98]. First, promoters tethered near a cluster fire more often than distant ones (in Fig. 3Aii, *e* fires more than *d*). Second, different clusters contain different TFs specializing in transcribing different small-world groups of transcription units (in Fig. 3Aii, pink housekeeping units co-fire in the pink cluster, and green units firing only in neurons co-fire in the green cluster). Consequently, neurons and fibroblasts contain different clusters transcribing different units; both also contain common clusters making housekeeping mRNAs and eRNAs (manuscript submitted by the author entitled ‘Transcription clusters and developmental pathways: nature, nurture, noise’). In other words, cluster type determines cell fate. Of course, epigenetic inputs and signals from the surroundings reinforce particular fates [99].

As evidence for this alternative (Fig. 3Aii) has been reviewed [91, 98], I mention just three kinds supporting it. First, most genomic contacts should involve active transcription units; they do. Thus, in bacteria, Hi-C shows that active RNA polymerases anchor essentially all loops [100]. In mammals, the highest-resolution contact data available (obtained using region-capture micro-C and the *Klf1* + *Ppm1g* loci in mouse ES cells) reveals that 67%–74% contacts involve active units making mRNAs and eRNAs (contrast 4% involving CTCF and cohesin) [101]. Second, essentially all transcription should occur in clusters; it does. Thus, our genome encodes hundreds of rRNA genes, but only those clustered in nucleoli are copied by polymerase I [102, 103]. Similarly, >92% of all nascent RNAs made by polymerases II and III are concentrated in extra-nucleolar clusters [93]. Third, related units should be co-transcribed in clusters rich in appropriate TFs; they are. Thus, gene-expression networks are small-world ones [104, 105], with immediate-early genes activated by NFκB, and other gene sets by ERα (or KLF1, or TFEC, or etc.) all co-clustering only when active [94, 106–108].

I describe clusters as ‘red’, ‘green’, or ‘blue’ to represent those found in different cell types. However, in practice, colors blend in complex ways [109], and this is reflected in single-cell transcriptomes where each cell in a population of fibroblasts, for example, has its characteristic transcript pattern [35, 37].

### Forces driving clustering

Three important forces drive clustering. First, a classical mechanism involves TFs able to dimerize, as many do (Fig. 3Bi) [110]. Thus, once two TFs bind to nearby cognate sites, they are transiently trapped in a local volume, and this increases the chances they collide and dimerize. Second, consider a DNA-binding protein/complex of >5 nm that has no affinity for any other protein (Fig. 3Bii). In a crowded cell, many small macro-molecules (<5 nm) bombard it from all sides. If two large molecules happen to touch each other, small ones are sterically excluded from entering the green volume between the two and so cannot knock them apart. Consequently, small molecules exert a force on opposite sides to keep the two large ones together—even though they have no affinity for each other. In other words, free-energy costs of ordering the two large molecules are less than the benefits accruing from the increased disorder of many small molecules. If complexes bind to DNA, this entropic ‘depletion attraction’ stabilizes clusters [111]. A third force was uncovered using (Brownian dynamics) simulations of red spheres (representing large polymerase/TF complexes) binding reversibly to promoters; spheres spontaneously clustered (Fig. 3Biii) [112, 113]. This emergent process requires bi-/multi-valency, so a complex can act as a bridge to stabilize a loop; consequently, it is called a ‘bridging-induced attraction’. Forming a bridge doubles the local concentration of promoters, which triggers positive feedback that operates without extra energy input to recruit more complexes/promoters (up to a limit where it becomes impossible to pack in more DNA). When red and green complexes are simulated, distinct red and green clusters emerge. When strings representing whole human chromosomes are colored according to local activity, topologically associating domains and A/B compartments all appear without invoking additional mechanisms. Given these three forces, Nature has a choice: either expend energy to stop clustering, or go with the flow and exploit it—as we suggest [98].

### Cluster stability and memory of cell fate

Cell fate is generally seen to be specified through the operation of gene regulatory networks coupled to epigenetic marking of chromatin [114]. Simulations of networks with randomly connected nodes show that islands of stability (‘attractor’ states) emerge naturally [115]. However, biological networks are wired specifically and hierarchically: master regulators control minor ones, and eventually effector genes through positive and negative feedback [116]. The alternative view of transcription points to an additional and stable way of encoding cell fate that works through clusters defining cell type. These defining networks are physical structures (contrast abstract gene-regulatory networks). I now describe how such networks change during development.

**Figure 4.**
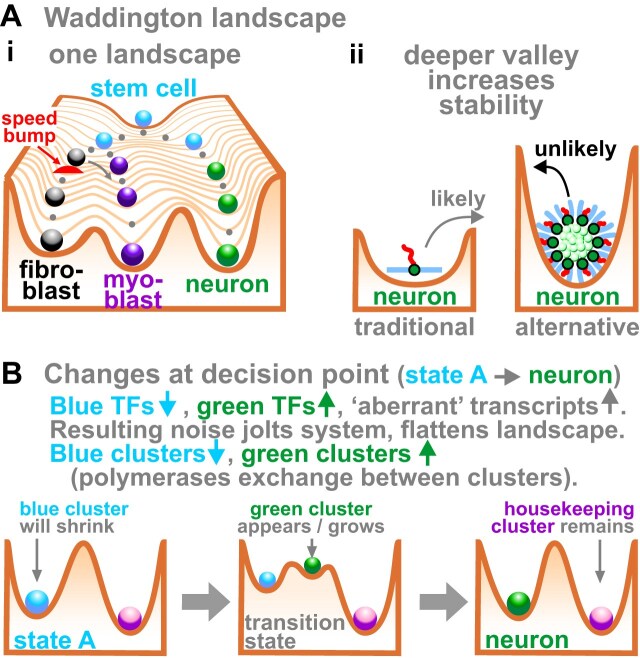
Clustering increases stable expression. (**A**) Waddington landscapes (iconography adapted from [117, 119]) . **(i)** A stem cell (blue ball) differentiates as it rolls down through a landscape. Transcriptional or epigenetic noise (represented as a red speed bump) might divert a cell down the wrong track (gray arrow). (**ii**) Clusters are in deeper valleys (lower energy potential) than singletons, and this mitigates the effects of noise inadvertently changing cell fate (e.g. by switching a neuroblast into a myoblast path). (**B**) Noise flattens the landscape to facilitate differentiation by reducing the activation energy required to pass over ridges to reach new states. Clusters now shown as spheres.

Consider a ‘Waddington landscape’ where ‘hills’ and ‘valleys’ represent ‘energy potentials’ guiding cells toward desired fates (Fig. 4Ai) [117, 118]. Inevitable transcriptional or epigenetic noise is visualized as a ‘speed bump’ that appears randomly to divert a cell down the wrong path (here, the bump diverts the progenitor down the unwanted myoblast path) [119]. According to any model, the system is more robust if valleys are deep, so cells are less likely to pass inadvertently into the wrong valley (Fig. 4Aii). We have seen that entropic forces drive singletons into clusters (Fig. 4B), with experiments confirming this [93], so the balance of forces must ensure that clusters are in deeper free-energy minima than singletons (manuscript submitted by the author entitled ‘Transcription clusters and developmental pathways: nature, nurture, noise’). Consequently, transcribing clustered genes embedded deep in Waddington landscapes should lessen the effects of noise, to stabilize clusters and enhance fate memory.

Cells rolling through the landscape pass decision points determining fate. Remarkably, cells at these points over-express apparently unwanted transcripts [120, 121], which is captured by this title: ‘*Transcriptome-wide noise controls lineage choice in mammalian progenitor cells*’ [121]. I suggest expressing new TFs increases noise, jolts the system close to chaos [21], and flattens the landscape to ease transition to a new state (Fig. 4B; manuscript submitted by the author entitled ‘Transcription clusters and developmental pathways: nature, nurture, noise’). Then, reprogramming fibroblastic cell fate—into myoblasts by transfecting a complementary DNA (cDNA) encoding MyoD [122], or into induced pluripotent cells using four Yamanaka factors (Oct4, Sox2, c-Myc, and Klf4) [123], or into neurons using chemicals [124, 125]—would all act by flattening landscapes.

Many inputs influence fate decisions and stabilize choices; these can act stochastically, inductively, and selectively [126–128]. For example, grafting experiments in mouse embryos decisively show that naïve cells can be forced to develop along quite different pathways [127, 128]. Moreover, many epigenetic modifications act at the level of transcription (and so in clusters) to establish and reinforce choices (e.g. through histone/DNA methylation allied to ‘imprinting’) [114, 118, 129–131]. Note that all these inputs act slowly.

**Figure 5.**
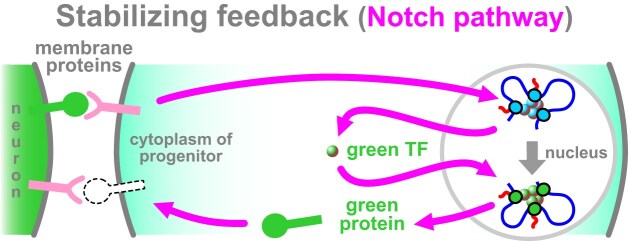
During development, positive feedback (slowly) reinforces fate choices. Here, a neuronal stem cell expressing a purple membrane protein detects an appropriate contact with a neuron (left); this activates the Notch pathway (purple arrows). Genes encoding neuronal TFs are now transcribed in blue clusters, and new TFs (green) made. In turn, these TFs drive co-clustering and co-transcription of neuronal genes (green), and some of the resulting neuronal proteins (green) are incorporated into the membrane to reinforce contacts with the neighboring neuron.

Signals from adjacent cells also influence fate choices, which I illustrate using the Notch pathway—chosen because it plays a critical role in long-term memory from worms to mammals [132–134], and because it ‘talks’ to other pathways influencing memory processing like the Wnt one [135]. Imagine a progenitor cell sits next to a mature neuron, and contact stimulates the pathway (Fig. 5). Signals from membrane receptors (Notch1-4 in mammals) to nuclei switch on expression of fate-determining TFs and associated epigenetic marks that trigger synthesis of new membrane proteins reinforcing contacts with neighbors. As information flows take many minutes, this is useful for fate consolidation.

### DNA: a nano-wired computer/memory system determining cell fate

**Figure 6.**
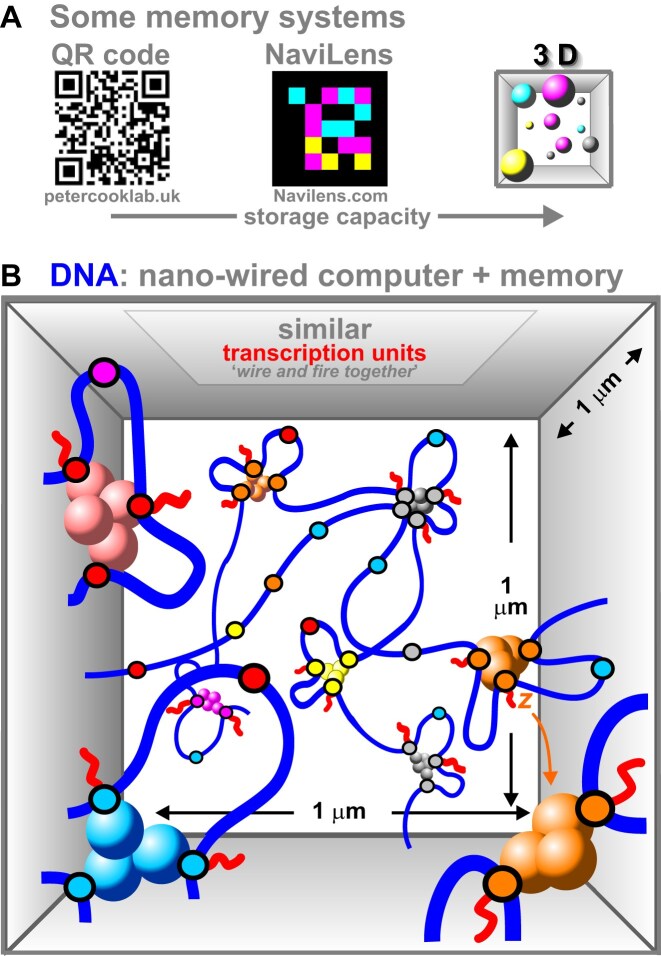
Some memory systems. (**A**) Examples. The 2D arrangement of black and white pixels in a QR code store information. Colored pixels increase capacity in NaviLens arrays. A third dimension should increase capacity further (pixels now shown as spherical ‘voxels’). (**B**) Cartoon illustrating part of the DNA computer/memory system in a human fibroblast (adapted from [172]). DNA folding ensures appropriate similarly colored promoters (small spheres) wire and fire together in similarly colored clusters. Cluster density (∼9/µm^3^) is roughly correct [171, 173], but only a few loops with a few active promoters are shown for clarity (there are ∼10 loops/cluster) [98]. It is unlikely the same set of clusters is found in any other fibroblast in a clone as there are so many potentially active promoters (compared to currently active ones), and so many possible similarly colored clusters. When transcription of *z* terminates, *z* might later be transcribed in a different cluster (bottom right).

Quick-response (QR) codes with black and white pixels provide high-density ways of storing information; Navilens arrays increase capacity by adding colored pixels, and 3D arrays should increase capacity further (Fig. 6A). Chromosomes in each cell in our body constitute a related 3D memory mechanism ensuring appropriate transcription units ‘*wire and fire together*’ (Fig. 6B). In other words, chromosomes act as nano-wired computer/memory systems. One task of this computer is to ‘remember’ cell fate, and—by analogy to an engram—I will call its ‘memory’ traces ‘transcriptional engrams’ (tengrams). Note, that a tengram is stored in a set of clusters in one nucleus (not in a group of cells). Then, the 1D genomic positions of TF-binding sites—which are highly conserved [136, 137]—dictate where TFs bind and cluster. Entropic forces allied to DNA flexibility then fold the genome into one of a countless number of possible 3D structures, with each encoding a slightly different kind of fibroblastic or neuronal fate (think of QR codes specifying different ‘Bakery’ or ‘Frozen’ items in supermarkets).

What logical symbols might this computer use, and how are they processed? I speculate they will be more numerous than those in the genetic code (which include A/C/G/T, triplet/start/stop codon, open-reading frame, promoter) as we should add ones representing 1D positions of binding sites for TFs, the concentrations of bi-/multi-valent TFs and their affinities (which, in turn, determine cluster color/number/type). Algorithms running on this computer must also add, subtract, and integrate symbols over time to generate circadian rhythms.

## Specialized transcriptional clusters and calcium signaling were probably in the first cells

The first nervous systems may have evolved independently more than once in radially symmetric metazoans (e.g. corals, sponges) and bilaterians [138]—and perhaps even before metazoans emerged [139]. They probably allowed their possessors living in water to engulf food (in corals) or move towards it (in bilaterians) [140, 141]. As evolution often tinkers with pre-existing systems, what information storage/transfer systems might have been available at the time?

**Figure 7.**
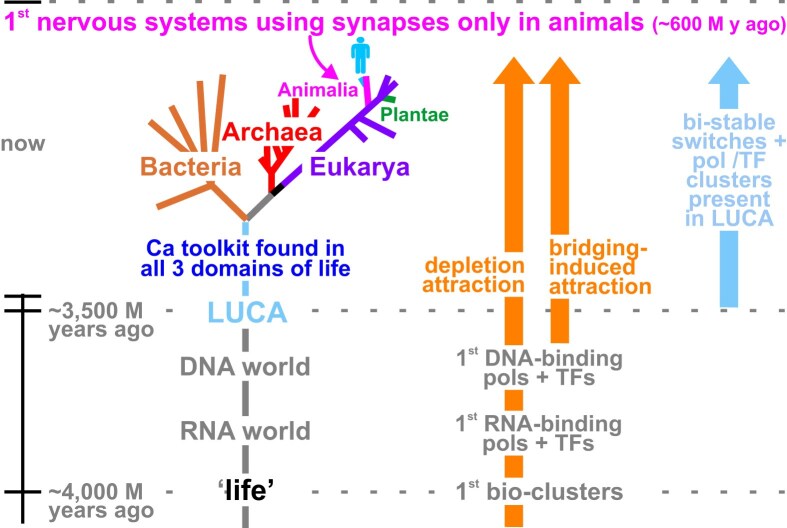
A simplified tree of life rooted in the RNA/DNA worlds, showing when the calcium toolkit evolved, when the first nervous system(s) using synapses appeared in animals, and some forces driving clustering. Clusters of polymerases (pols) + TFs, and biphasic switches containing kinases, were probably present in LUCA. Adapted from https://en.wikipedia.org/wiki/Last_universal_common_ancestor (accessed 31 January 2024), and https://www.petercooklab.uk/pan-genomic-model/8-faqs (accessed 9 March 2026).

An RNA world probably preceded DNA-based LUCA (the Last Universal Common Ancestor) from which all bacteria, archaea, and metazoans are descended (Fig. 7) [142]. Phylogenetic comparisons indicate LUCA likely encoded kinases, polymerases and TFs related to those we know today [143]. As entropic processes like the depletion and bridging-induced attractions act throughout time, they would have driven the first polymerases and TFs into clusters, and—once different TFs emerged—clusters specializing in transcribing different gene sets (Cook PR. The pan-genomic model: 8 FAQs. https://www.petercooklab.uk/pan-genomic-model/8-faqs accessed on 9 March 2026). Consequently, LUCA probably contained kinase-based bivalent switches and differently colored clusters enabling it to grow or shut down depending on the availability of food. What is known as the calcium toolkit probably then evolved, as it is found in all three domains of life (Fig. 7) [139]. This toolkit enables Ca transients to cross cells in <1 s [43, 44, 144]. Much later, when the first nervous systems using synapses evolved, I suggest Nature exploited these tried-and-tested storage and signaling modules. Of course, many other mechanisms are needed to allow such nervous systems to evolve (e.g. those facilitating vesicular traffic, sodium ion flows, etc.) [139].

## A transcription-centric view of memory engrams

**Figure 8.**
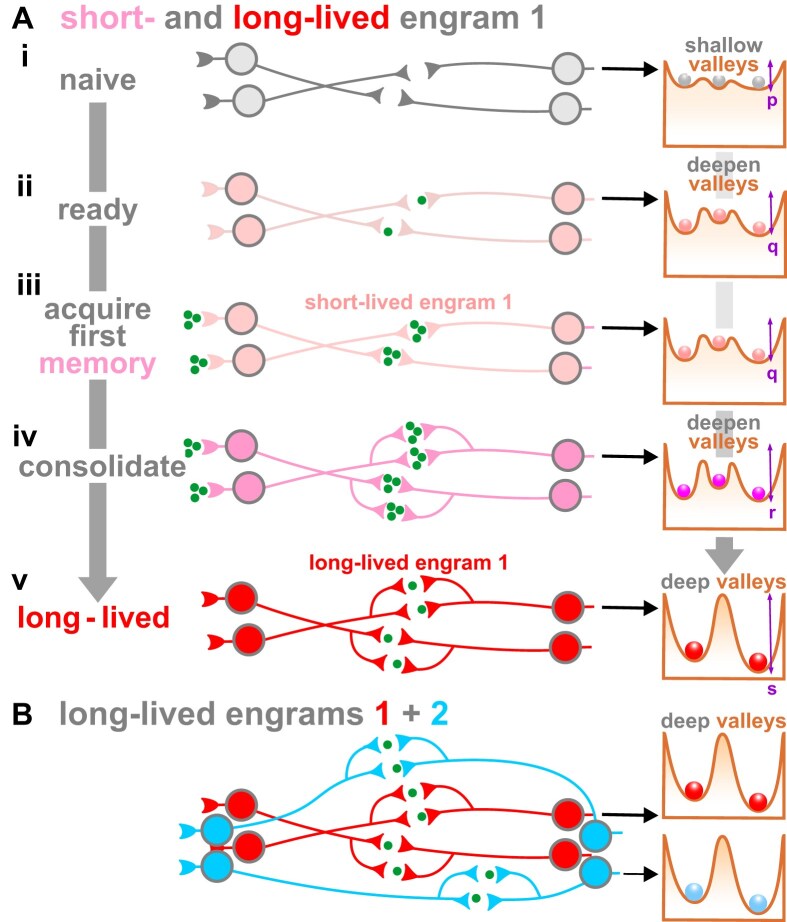
A transcription-centric view of engrams (iconography adapted from [12]). Chemical information flows at synapses: green dots. Waddington landscapes and relevant clusters in one neuron shown on right (valley depth p < q < r < s, valley deepening is slow and requires transcription). (**A**) Development of engram 1. **(i)** Four naïve neurons with projecting axons. Noisy transcription ensures clusters are in shallow valleys. **(ii)** These neurons happen to contain the same kind of (pink) clusters so their cell surfaces possess compatible binding proteins. They begin to fire spontaneously at random—and so ‘fire and wire’ together. Feedback into nuclei stabilizes transcription in clusters (so clusters are now in slightly deeper valleys), and newly made proteins stabilize synaptic connections. **(iii)** During acquisition of the first engram, chemical information flows rapidly through synapses (valley depth unchanged). **(iv)** Slow consolidation involves further feedback that deepens valleys and stabilizes synaptic connectivity. **(v)** Transition to an enduring memory requires more noise reduction, deepening valleys, and further transcription plus translation. A reverberating circuit is now maintained by low levels of synaptic traffic. Tengrams are responsible for creating the initial physical substrate, and transcription plus translation maintain enduring engrams through valley deepening. (**B**) A second (different) long-term memory is stored in cells with different (blue) clusters in deep valleys.

Against this background, I rephrase the standard model as follows. Our naïve neurons (Fig. 8Ai) contain potential engram cells that can be classified by their type of transcriptional cluster. Spontaneous spiking prior to our first memory pre-connects neurons with compatible surface molecules specified by similar clusters (pink here). These clusters exist in a Waddington landscape in a noisy transition state. Repetitive co-firing activates pre-existing immediate-early proteins like c-FOS, and these alter nuclear transcription (and so the landscape) to slightly deepen valleys containing pink clusters. The resulting naïve Hebbian circuit is now ready (Fig. 8Aii) to acquire its very first memory (probably of our mother; Fig. 8Aiii) that will be stored as a ‘pink’ engram in the most accessible of these networks (e.g. ones containing pink clusters). Consolidation strengthens co-firing synapses, adds dendrites/spines/nanotubes, and slowly deepens valleys (Fig. 8Aiv). Subsequent memories associated with our mother are allocated to this pink group, as well as overflowing to include new cells with some shared receptors (to give engrams with darker shades of pink). If our mother sees our survival is at stake, her scream is so loud the memory is directed straight into one of these networks, and she hopes we take appropriate action (recall the lab mouse scurrying for shelter).

Now a memory is stabilized: noisy transcription falls, and the pink network differentiates (needing new transcription and translation) into a (red) one with synaptic connections stable enough to last a lifetime (Fig. 8Av). Other engrams associated with our mother would be stored in cells with reddish blends of clusters. When our mother again sees our survival is at stake, her scream ensures we really get the message, and a fraction of this red network now stores an apparently inherited ingram (actually an acquired memory that triggers the response of ‘freeze’ or ‘run back home’). Engrams of a different color store memories unrelated to our mother (blue in Fig. 8B).

In principle, this transcription-centric view—which lies centrally within the standard model—can be tested by monitoring whether new clusters appear and disappear as new memories are acquired and lost. In practice, this is difficult for three inter-related reasons. First, transcriptomics focuses on mature mRNAs, not nascent RNAs. Second, nascent mRNAs represent the minority of nascent transcripts, as the majority are nascent eRNAs. Third, genome-wide association studies show that thousands of loci (mainly ones encoding eRNAs, not proteins) control cell fate (manuscript submitted by the author entitled ‘Transcription clusters and developmental pathways: nature, nurture, noise’). Consequently, we need to broaden focus from a few mRNAs to thousands of nascent non-genic RNAs to test this variant of the standard model.

## Speculation: DNA computers store and manipulate our neural memories

**Figure 9.**
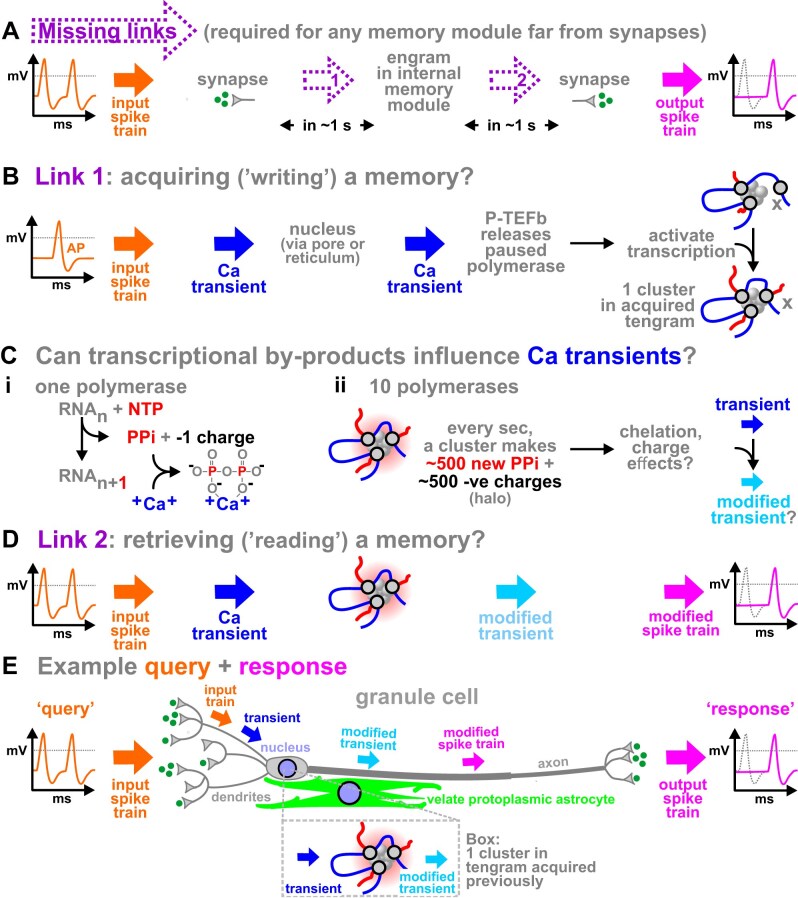
Speculative pathways for storing neural memories in DNA computers. (**A**) Two missing links. When an input spike train reaches a synapse, link 1 is required to ‘write’ an engram/tengram, and link 2 to ‘read’ the information and generate an output—both in ∼1 s. (**B**) Link 1: tengram acquisition. An action potential (AP) induces a Ca transient that passes through the nuclear pore/reticulum to release P-TEFb brakes pausing polymerases and remodel clusters (here, two polymerases restart and x attaches and fires). A set of remodeled clusters represents a tengram storing the neural memory. (**C**) The transcription reaction. **(i)** When a polymerase adds a nucleotide, one PPi is released with a net charge change of −1. One PPi can chelate one Ca^2+^ ion. **(ii)** Ten polymerases generate more PPi and negative charges (values calculated assuming polymerization at 50 nucleotides/s). Can chelation/charge effects modify passing transients? (**D**) Link 2: tengram retrieval. Can a spike train create transients that are modified as they pass through local concentrations of PPi, and which then alter a spike train so it now carries a retrieved memory? (**E**) Example. Imagine the system queries (spike chain on left) whether a granule cell contains a neuronal memory stored as a tengram (part shown at center). After the input synapse, the action potential (left-hand gold arrow) generates a transient (dark blue arrow) that sweeps past the nucleus to be modified by local concentrations of PPi/charge. The now-modified transient (light blue arrow) alters the spike train (right-hand purple arrow)—which carries the response (spike train on right) to output synapses and beyond. The granule-cell nucleus is so positioned it receives inputs from many synapses (in ‘claws’ on 3–5 dendrites) within ∼1 s, so it can integrate different inputs). It also fills most of the cytoplasm (leaving a surrounding rim ∼50 nm thick that rarely contains endoplasmic reticulum [174–176]. Therefore, a passing transient could be modified by PPi/charge clouds (box). Here, an astrocyte ‘veil’ embraces the granule-cell soma to electrically connect (via transients) DNA computers storing tengrams in different neuronal and non-neuronal nuclei.

Now consider a radical addition to the standard model in which some—perhaps all—long-term memories are stored and manipulated in the DNA computers that specify cell fate. Imagine we are asked when we were born. Although we might have learned the answer a long time ago over days or weeks, we can respond in a second. Consequently, information must flow quickly through what I called the two ‘missing links’—in this case from a synapse to the nuclear tengram containing our date-of-birth, and from there to the appropriate output synapse (Fig. 9A). I now suggest plausible mechanisms for each link.

Nucleoplasms of all cells (including hippocampal neurons) [145] contain the Ca toolkit so that transients accompanying depolarization can pass in <1 s from the cell membrane to the nucleoplasm via nuclear pores or the nuclear reticulum (Fig. 9B, left) [45, 146, 147]. Note that transients can travel bi-directionally in neurons [148], and that ‘*The requirement of nuclear calcium signals for long-term memory formation is evolutionarily conserved from flies to mammals*’ [43]. Tengram acquisition could then occur as follows (Fig. 9B, right). In non-neural cells, a significant fraction of RNA polymerase II is paused because a critical elongation factor (P-TEFb) is sequestered [149, 150], and a transient can immediately release the pause (via calmodulin, PP2B/calcineurin, 7SK snRNP, and CDK9) [151, 152]. In neuronal cells, another player in this circuit—HEXIM1—sequesters P-TEFb and regulates immediate-early gene transcription [153]. Therefore, there are ample precedents for all steps in the first missing link: transients rapidly change transcription, and so the pattern of nuclear clusters (Fig. 9B).

Consider the second missing link. When a productively elongating RNA polymerase II adds a nucleotide, one pyrophosphate (PPi) is released as the net charge changes by −1 (Fig. 9Ci). Importantly in this context, PPi is a powerful Ca-chelating agent (the apparent *K_d_* of ∼1 µM is highly dependent on local ion/charge concentrations). It is quickly cleaved into two monophosphates (giving more charge), and charges neutralized. Since human RNA polymerase II adds ∼50 nucleotides/s, a cluster generates ∼500 PPi + ∼500 negative charges every second (Fig. 9Cii). These numbers probably represent lower bounds, as non-productive enzymes make short soon-to-abort transcripts that go undetected using most current methods ([154]; Cook PR The pan-genomic model: 8 FAQs https://www.petercooklab.uk/pan-genomic-model/8-faqs, accessed on 9 March 2026). Additionally, rDNA clusters in HeLa cells contain ∼500 active polymerase I enzymes [155, 156], so nucleolar clusters generate much larger numbers. Note that Ca induces calmodulin to bind to the DDX21 helicase—which normally sequesters the catalytic subunit of polymerase I (RPA194), so the free enzyme can transcribe [157]. Can local Ca concentrations be detected in nuclei? Remarkably, they can: Ca imaging uncovers discrete foci in cardiomyocyte nuclei (Jakub Tomek, personal communication) that are reminiscent of those containing nascent RNA [92]. Additionally, local charge densities outside neurons affect spike transmission within neurons [158], so could internal densities do so too? I suggest the system exploits Goldilocks concentrations of PPi plus charge to modify the shape of (or destroy) transients (Fig. 9Cii, right) [159]. Consequently, an input spike train (essentially a series of digital signals) generates a modified transient (an analog signal) that—in turn—feeds back to modify the train (Fig. 9D). In this example, the system reduces wave height. Strikingly however, cardiomyocyte nuclei can generate new waves; moreover, in bi-nucleate cells in the population, a random pattern of wave creation can settle into an alternating (oscillatory) one (Jakub Tomek, personal communication). This demonstrates that these nuclei contain the necessary reaction-diffusion machinery for generating waves (which I suggest is bound to clusters), and that different nuclei can inter-communicate.

Now imagine the system queries whether a particular granule cell contains our date-of-birth, written as a tengram in clusters that are a whiter shade of pale. Then, a ‘query’ depolarization generates transients that pass through the nucleoplasm where they are modified to give the ‘response’ (Fig. 9E). If now asked ‘where were we born?’, one can imagine this computer slowly processes related data at ∼10 bits/s.

**Figure 10.**
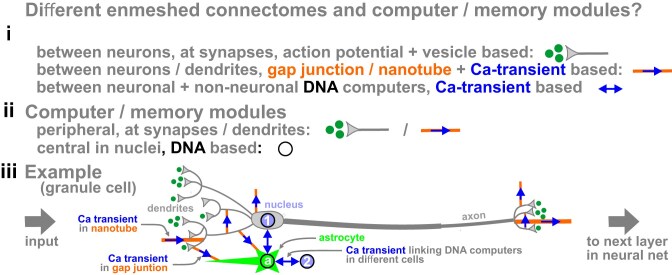
Different connectomes and computer/memory systems in neural nets in our brains. **(i)** Connectomes. The first is as in the standard model, and the second includes transient-based signaling through gap junctions and nanotubes [177]. The third adds transient-based signaling between neuronal and/or non-neuronal cells, as speculated here. **(ii)** Computer/memory systems. The first is as in the standard model, and the second is speculated here. **(iii)** Example cell in one layer in our net. Here, a DNA computer in granule cell 1 is linked with others in astrocyte a + granule cell 2. Cell 1 is connected on the left and right to other layers through the 1^st^ and 2^nd^ connectomes, and to astrocyte a through the 2^nd^ and 3^rd^. In other layers in our net, some astrocytes contact ~10^6^ synapses [178], and such connectivity can multiply computing power dramatically [179]; therefore, it is likely different layers exploit different connectomes/computers in different ways.

According to the standard model, both long- and short-term memories are stored and processed at synapses (through vesicular traffic) in neural nets in our brains (first possibilities in Fig. 10i and ii), and perhaps through the supporting calcium-based system via gap junctions plus nanotubes electrically linking astrocytes/glia/etc. (Fig. 10i, second possibility). This radical view adds a third connectome, where transients link nuclei in neuronal as well as non-neuronal cells (Fig. 10i, third possibility), and adds DNA computers to synaptic ones (Fig. 10ii). Recall that the first nervous systems utilized Ca-based information transfer, with vesicle-based systems only evolving later (Fig. 7).

This view raises many questions, for example: (i) How many enduring memories are stored in DNA computers compared to synaptic ones (and in neuronal cells versus non-neuronal ones)? (ii) How do connectomes vary in different layers in the net, and how does the system allocate queries to different ones? (For advantages and disadvantages of intra-layer versus pipeline parallelism used in large language models, see [160], and for associated scheduling, see [161].) (iii) Do clusters contain the Ca toolkit, and can some act as Ca sources as well as sinks (recall cardiomyocyte nuclei generating new oscillatory waves)? (For ways of partially purifying clusters from non-neuronal cells and analyzing their content, see [162, 163].) Then, Ca-mediated reaction-diffusion could allow communication between different clusters/addresses within one DNA computer. (For ways that random cytoplasmic distributions of Ca sources/sinks facilitate emergent behaviors like signal amplification and percolation, see [164, 165].) (iv) What new behaviors does communication between different DNA computers bring to the net? For example, can the system exploit the many advantages of oscillatory computing [166–168] that include ultra-low energy operation, massive parallelism, rich representational capacity through variable phase/frequency, and noise robustness?

This radical view can be tested by seeing how transcriptional inhibitors affect passing transients and spike trains. However, disentangling Ca-specific effects will inevitably be difficult given that ‘*All cells use Ca^2+^ signals to regulate their activities in response to extrinsic and intrinsic stimuli*’ [44].

## Conclusions

Many have addressed how memories endure for decades, and various reasons embolden me to add to the cacophony. First, when compared with other cell types, brain cells are transcriptionally hyper-active and make many apparently noisy transcripts. Second, there are two well-known molecular mechanisms able to ensure other kinds of memory can last a lifetime. One involves changing nucleic-acid sequence, but there is no widely accepted evidence for directed changes in neuronal DNA or RNA when we remember and forget. The other is the way that progeny of fibroblasts ‘remember’ their cell fate (Fig. 2C), so it seems worth considering what enables this. Third, there is increasing support for an alternative view of transcription where clusters of RNA polymerases co-transcribe functionally related genes determining cell fate (Fig. 3Aii). In other words, these clusters are nano-wired DNA computers enabling different sets of transcription units to wire and fire together (Fig. 6). Fourth, forces driving clustering act throughout time, so clusters probably existed in the very first cells (Fig. 7). Therefore, I suggest clusters would be tested for their suitability as memory modules when the first nervous systems evolved. These reasons lead me to reinforce a transcription-centric view of the standard model where clusters play critical roles at every stage in the life of an engram (Fig. 8): they define which cells are included in Hebbian circuits (through possession of compatible surface molecules), and provide mechanisms for consolidation and storage of long-term memories (by slow cell ‘differentiation’ and valley deepening). Processing long-term memories would use strengthened synapses with algorithms running in the complex inter-leavings of neural-cell connectomes [169, 170]. I also speculate that DNA computers specifying cell fates can also store our memories as tengrams (Fig. 9), with inter-leavings of different kinds of computers and connectomes providing the rich complexity needed to store and process our enduring memories (Fig. 10).

In summary, I suggest DNA stably encodes three kinds of information. First, the base sequence specifies transcript sequence, and so protein sequence. Second, bound TFs self-assemble into transcriptionally active clusters that determine cell fate and tissue organization. Third, I speculate these clusters also store and process our enduring memories.

## Data Availability

No new data were generated or analysed in support of this research.
